# A system for production of defective interfering particles in the absence of infectious influenza A virus

**DOI:** 10.1371/journal.pone.0212757

**Published:** 2019-03-01

**Authors:** Najat Bdeir, Prerna Arora, Sabine Gärtner, Markus Hoffmann, Udo Reichl, Stefan Pöhlmann, Michael Winkler

**Affiliations:** 1 Infection Biology Unit, German Primate Center–Leibniz Institute for Primate Research, Göttingen, Germany; 2 Faculty of Biology and Psychology, University Göttingen, Göttingen, Germany; 3 Max Planck Institute for Dynamics of Complex Technical Systems, Bioprocess Engineering, Magdeburg, Germany; 4 Otto von Guericke University Magdeburg, Chair for Bioprocess Engineering, Magdeburg, Germany; University of Iowa, UNITED STATES

## Abstract

Influenza A virus (IAV) infection poses a serious health threat and novel antiviral strategies are needed. Defective interfering particles (DIPs) can be generated in IAV infected cells due to errors of the viral polymerase and may suppress spread of wild type (wt) virus. The antiviral activity of DIPs is exerted by a DI genomic RNA segment that usually contains a large deletion and suppresses amplification of wt segments, potentially by competing for cellular and viral resources. DI-244 is a naturally occurring prototypic segment 1-derived DI RNA in which most of the PB2 open reading frame has been deleted and which is currently developed for antiviral therapy. At present, coinfection with wt virus is required for production of DI-244 particles which raises concerns regarding biosafety and may complicate interpretation of research results. Here, we show that cocultures of 293T and MDCK cell lines stably expressing codon optimized PB2 allow production of DI-244 particles solely from plasmids and in the absence of helper virus. Moreover, we demonstrate that infectivity of these particles can be quantified using MDCK-PB2 cells. Finally, we report that the DI-244 particles produced in this novel system exert potent antiviral activity against H1N1 and H3N2 IAV but not against the unrelated vesicular stomatitis virus. This is the first report of DIP production in the absence of infectious IAV and may spur efforts to develop DIPs for antiviral therapy.

## Introduction

Influenza A virus infection is responsible for annual influenza epidemics and intermittent pandemics that are associated with significant morbidity and mortality [1]. The ability of IAV to constantly change in response to immune pressure or antiviral treatment limits the effectiveness of currently used antiviral interventions. Thus, vaccines against seasonal influenza need to be annually reformulated and will provide little if any protection against pandemic influenza [1]. Moreover, the effectiveness of antivirals targeting the viral proteins M2 and neuraminidase is compromised by the frequent emergence and transmission of resistance mutations [1, 2]. Therefore, novel approaches to combat influenza are urgently needed.

IAVs are enveloped and harbor eight segments of genomic viral RNA. Defective interfering (DI) genomic segments can be generated in IAV infected cells due to errors of the viral polymerase [3, 4]. DI segments usually harbor a large deletion which inactivates the open reading frame encoded by the segment [3, 4]. The DI segments can interfere with amplification of wild type (wt) segments, potentially by competing for viral and cellular resources required for segment replication. Moreover, DI RNAs can be packaged into progeny virions, termed defective interfering particles (DIPs), and coinfection of target cells with DIPs and IAV will result in preferential amplification of DIPs and suppression of IAV spread [3, 4]. This effect has been observed in cell culture [5–8] and in experimentally infected animals [5, 9–15] and may extend to unrelated viruses [14, 16], due to the activation of the interferon system [15, 16]. Moreover, DIP application in a therapeutic or preventive setting prevents or ameliorates influenza in animal models [3–5, 10–16]. In sum, DIPs can be considered natural antivirals produced in the context of infection with IAV and many other viruses and may provide a basis for the development of new strategies for antiviral intervention.

At present, amplification of DIPs requires coinfection of cells with DIPs and wt virus, termed standard or helper virus, which subsequently needs to be inactivated by UV light [3, 4, 17, 18]. The presence of standard virus poses a safety concern when products for animal and human use are generated and complicates the interpretation of experimental data. Plasmid systems encoding for wt and DI segments along with cell lines expressing the IAV proteins for which the genomic information has been lost upon DI RNA formation might circumvent this issue [4, 19]. However, expression of the viral polymerase subunit PB2 in trans was found to be insufficient for robust amplification of IAV variants harboring temperature sensitive mutations [20, 21] and it has been speculated that similar limitations might apply to the production of DIPs [4]. Moreover, it has been suggested that PB2 expression might be toxic to cells [4]. Therefore, it is currently unknown whether the strategy outlined above might allow for production of segment 1-derived DIPs and at present no system for generation of DIPs in the absence of standard virus has been reported.

DI-244 is a naturally occurring DI-RNA found in hen’s eggs [22]. DI-244 is derived from segment 1, which encodes PB2, and harbors a 1,946 nucleotides comprising deletion [4, 22]. This deletion removes most of the PB2 ORF but leaves the 3’ 244 nucleotides and 5’ 151 nucleotides of segment 1 intact which are sufficient for segment replication and packaging [4, 22]. Here, we investigated whether coexpression of wt segments 2–8, PB2 protein and DI-244 RNA allows for production of DIPs. Employing a novel DI-244 variant encoding mScarlet-i, we show that DI-244-based DIPs are efficiently produced in cells expressing a codon optimized version of PB2 and that these DIPs exert potent antiviral activity.

## Material and methods

### Plasmids and oligonucleotides

Plasmids for rescue of the A/PR/8/34 (H1N1) strain, pHW191-pHW198, were used throughout this study and have been previously described [23]. To generate a retroviral vector encoding PB2, the PB2 open reading frame was amplified from pHW191 using primers PB2-QCXIP-5N (5- CCGCGGCCGCACCATGGAAAGAATAAAAGAACTAC-3) and PB2-3XBgl (5-GGAGATCTCGAGCTAATTGATGGCCATCCGAAT-3) and cloned into the retroviral vector pQCXIP-mcs using NotI and XhoI [24]. This self-inactivating vector allows constitutive expression of PB2 and puromycin resistance genes coupled by an internal ribosome entry site (IRES). An optimized sequence of PB2 was generated by hand to maximize sequence deviation from PB2 and optimizing codon usage for influenza A virus and humans (S1 Fig). This sequence was synthesized and cloned by GeneArt (Regensburg, Germany) and subcloned using NotI and XhoI sites into pQCXIP-mcs. A plasmid for DI-244 rescue was generated by splice overlap PCR, using pHW191 as template and primer pairs fluA AarI-PB2-1G (5- CGATCACCTGCTCGAGGGAGCGAAAGCAGGTC-3)/IAVseg1-DI244rep-rev (5- AATGAGGAATCCCCTCAGTTAAGCGGCCGCTGCGGTACCAGATCTCTTCTCCTGTCTTCCTGA-3) and IAVseg1-DI244rep-for (5- TCAGGAAGACAGGAGAAGAGATCTGGTACCGCAGCGGCCGCTTAACTGAGGGGATTCCTCATT-3)/fluA AarI-PB1-2341R (5- CGATCACCTGCTCTCTATTAGTAGAAACAAGGCATTT-3). The product of the splice overlap PCR was then purified and amplified with the segment specific primer pair fluA AarI-PB2-1G/fluA AarI-PB1-2341R and cloned into pHW2000-GGAarI, using golden gate cloning, generating pHW2000-DI244-mcs [25]. In addition, a construct containing a multiple cloning site (mcs) was generated for later insertion of reporter genes. For this, the PCR fragments were amplified using pHW191 as template and primer pairs fluA AarI-PB2-1G/IAVseg1-DI244rep-rev (5- AATGAGGAATCCCCTCAGTTAAGCGGCCGCTGCGGTACCAGATCTCTTCTCCTGTCTTCCTGA-3) and IAVseg1-DI244rep-for (5- TCAGGAAGACAGGAGAAGAGATCTGGTACCGCAGCGGCCGCTTAACTGAGGGGATTCCTCATT-3)/ fluA AarI-PB1-2341R followed by splice overlap joining and golden gate cloning. As reporter gene, mScarlet-i without internal SalI and NotI sites and fused to the porcine teschovirus-1 (PTV1) 2A sequence (GATNFSLLKQAGDVEENPGP) was cloned into the mcs as a BglII/NotI fragment. In this way, a PB2 (aa 1–41)-2A-mScarlet-i ORF was generated, which allows the detection of the presence of DI-244 via mScarlet-i fluorescence. The template for mScarlet-i, pmScarlet-i_C1, was a gift from Dorus Gadella (Addgene plasmid # 85044) [26]. The integrity of PCR-amplified, cloned sequences was verified by sequence analysis.

### Cells and viruses

All cells were cultured at 37°C and 5% CO_2_. 293T human embryonic kidney cells and Vero cells were maintained in Dulbecco’s Modified Eagle Medium (DMEM; Gibco) containing 10% fetal bovine serum (FBS, Gibco), penicillin (Pen, 100 IU/ml) and streptomycin (Strep, 100 μg/ml). 293T cell lines stably expressing PB2 were grown in the presence of 1 μg/ml puromycin. Madin-Darby canine kidney cells (MDCK) were cultured in Glasgow’s MEM (GMEM) with 10% fetal bovine serum (FBS, Gibco) and Pen/Strep. All cell lines were obtained from collaborators and were regularly checked for mycoplasma contamination. MDCK cells stably expressing PB2 or PB2opt were cultivated in the presence of 1.5 μg/ml puromycin. Influenza A viruses A/Panama/2007/99 (H3N2) [24] and A/PR/8/34 (H1N1) produced in embryonated chicken eggs were used to assess the antiviral activity of DIPs. We further employed a recombinant vesicular stomatitis virus (VSV) that expresses a dual reporter consisting of eGFP and firefly luciferase from an additional transcription unit located between the open reading frames for the viral glycoprotein and polymerase [27].

### Production of retroviral vectors

The production of MLV particles for transduction of cells followed an established protocol [25, 28]. Briefly, 293T cells seeded in T25 flasks were transfected with 6 μg of retroviral vector (e.g. pQCXIP-PB2), 3 μg MLV-gag-pol plasmid and 3 μg VSV-G expression plasmid, employing the calcium phosphate transfection method. The culture medium was exchanged at 8 h after transfection. After 48 h, MLV particle-containing supernatant was harvested, cleared by passing through a 0.45 μm filter, aliquoted and then stored at -80°C.

### Transduction and selection of cell lines

For retroviral transduction, cells were seeded in 96-well plates at 5,000 (MDCK) or 10,000 (293T) cells/well in 50 μl cell culture medium. On the next day, 50 μl of supernatant containing MLV particles was added per well followed by spinoculation at 4,000 × g for 30 min for enhancement of transduction [29]. Two days after transduction, the cells were detached and transferred into 24-well plates containing cell culture medium supplemented with 1 μg/ml (293T) and 1.5 μg/ml (MDCK) puromycin. In parallel, non-transduced cells were treated similarly to control for effective cell killing by the antibiotics.

### Mini-replicon assay

293T were seeded at a cell density of 2 × 10^5^ cells/well in 12-well plates. The following day, the cells were transfected using the calcium phosphate method. The concentrations of plasmids to be transfected were largely adapted from published work [30]: 10 ng of pCAGGS plasmids encoding viral RNA polymerase proteins (PB2, PB, PA) and 100 ng of plasmid encoding NP were cotransfected with 50 ng of plasmid pPolI-Luc, which encodes the firefly luciferase reporter gene flanked by the noncoding regions of segment 8 of A/WSN/33. Empty plasmid was used to ensure that all transfections were conducted with the same total amount of plasmid DNA. For analysis of functionality of PB2 in 293T cells stably expressing this protein, transfection was carried out as described above but the plasmid encoding PB2 was omitted. As control, the plasmid encoding PB1 was omitted. The cells were washed at 6–8 h after transfection and harvested at 24 h post transfection. Luciferase activities in cell lysates were measured using the Plate Chameleon V plate reader (Hidex) and Microwin 2000 software.

### Immunoblot

For analysis of PB2 expression in 293T and MDCK cells, the cells were seeded in 6-well plates, incubated for 24 h, harvested and lysed in 200 μL of Laemmli SDS-PAGE sample buffer (5% glycerine, 1% SDS, 2.5% ß-mercaptoethanol, 0.5% Bromophenol blue, 0.5 mM EDTA,0.5M Tris pH 6.8). Samples were heated to 95°C for 10 min and separated via SDS-PAGE using 12.5% polyacrylamide gels. Proteins were then transferred onto a nitrocellulose membrane (GE health care) using a Mini-PROTEAN Tetra Cell (BioRad) powered at 110 V for 90 minutes. Membranes were blocked with 5% skimmed milk diluted in PBS-Tween and incubated with primary rabbit polyclonal antibodies against PB2 (1:1,000, GenTex, Irvine, USA) overnight at 4°C. Subsequently, membranes were washed and incubated with anti-rabbit HRP (horseradish peroxidase)-conjugated secondary antibodies (1:10,000, Dianova) for one hour. Finally, chemiluminescent substrate HRP juice plus (P.J.K.) was added onto the membrane and bands were visualized using a ChemoCam imager (INTAS). In order to detect ß-actin, the membrane was subsequently stripped using stripping buffer (62.5 mM Tris HCl pH 6.8, 2% SDS, 100 mM ß-mercaptoethanol) for 30 min at 5°C, washed three times with PBS-Tween, and incubated with anti ß-actin mouse (1:500 Sigma-Aldrich) overnight. The membrane was then washed and incubated with anti-mouse HRP-conjugated secondary antibody (1:10,000, Dianova) for one hour. HRP juice plus was added and bands were visualized as previously described. Quantification of PB2 and PB2opt expression was carried out using the program ImageJ (FIJI distribution) [31]. In order to normalize data, signals measured for PB2/PB2opt were divided by those measured for beta-actin.

### Production of defective interfering particles

For DIP production, a coculture of 200,000 MDCK cells and 700,000 293T cells stably expressing PB2 was seeded in T25 flasks. The next day, cells were cotransfected via the calcium phosphate method with 1 μg each of plasmids encoding DI-244-mScarlet-i and wt IAV genomic segments 2–8. Culture medium was changed at 8 h post transfection. At 48 h post transfection, cells were washed with phosphate buffered saline (PBS) without calcium and magnesium and DMEM medium supplemented with 0.2% BSA (MACS BSA), 0.5 μg/ml tosyl-phenylalany-chloromethyl-ketone (TPCK)-trypsin (Sigma), penicillin (100 IU/ml) and streptomycin (100 μg/ml) was added. As negative control, transfection of parental MDCK and 293T cells was analyzed. Supernatants were harvested from all cultures at 4, 6, 8 and 10 days post transfection, cleared by centrifugation at 4,000 rpm for 10 min to remove debris, aliquoted and stored at -80°C. Infectivity of supernatants was analyzed by focus formation assay as described [25, 32] but using MDCK cells expressing PB2 or PB2opt as targets. In brief, MDCK-PB2/PB2opt cells seeded in 96-well plates were washed and incubated for 1 h with serial dilutions of DIP-containing supernatants. Thereafter, supernatants were removed and infection medium (GMEM with 0.2% BSA and Pen/Strep) supplemented with 0.5% methylcellulose and 0.5 μg/ml TPCK-trypsin was added. Plates were incubated for 72 h and then stained using anti IAV polyclonal antibody (Millipore).

### Immunofluorescence

Images were taken on a Zeiss LSM800 equipped with a 10x/0.45 plan-apochromat objective, 488 nm and 561 nm diode lasers and ZEN imaging software (Zeiss). Fluorescent signals (red channel, 561 nm laser) were detected with GaAsP detector employing the same sensitivity for all images of a series, while bright field signals were recorded with an ESID detector (photodiode) with individually adjusted sensitivity.

### Analysis of antiviral activity of DIPs

To test antiviral activity of DIPs against IAV and unrelated VSV, we performed infection experiments in the presence of DIP-containing or DIP-free supernatants and subsequently compared viral titers in the culture supernatants. For this, MDCK cells were seeded in 96-well plates at a density of 10,000 cells/well. On the next day, DIP-containing supernatants or DIP-free control supernatants were 10-fold serially diluted. Subsequently, MDCK cells were washed twice with PBS and 50 μl of the respective supernatants were mixed with 50 μl of virus and the mixture inoculated onto the MDCK cells. After a 1 h incubation, 100 μl of fresh infection medium supplemented with 0.5 μg/ml TPCK-trypsin was added and the cells were further incubated for 24 h (VSV) or 72 h (IAV) before viral titers in the culture supernatants were determined. Virus titration was performed on confluent monolayers of MDCK (IAV) or Vero (VSV) cells that were grown in 96-well plates. After aspiration of the culture medium, cells were washed twice with PBS and inoculated with 50 μl of 10-fold serial dilutions of the culture supernatants of IAV or VSV infected MDCK cells. After 1 h of incubation with IAV containing supernatants, the medium was removed and 100 μl infection medium supplemented with 1% Avicel and 0.5 μg/ml TPCK-trypsin (IAV/MDCK) was added per well. After 1h incubation with VSV-containing supernatants, 200 μl infection medium supplemented with 0.5% methylcellulose (VSV/Vero) were added on top, and the cells were further incubated for 24 h. IAV titers were quantified by antibody staining, using the focus formation assay as previously described [25, 32]. In order to quantify VSV titers, eGFP-positive foci were counted under the fluorescence microscope. All titers are given as focus forming units per ml (ffu/ml).

## Results

### Generation of 293T and MDCK cells stably expressing functional PB2

**Fig 1:**
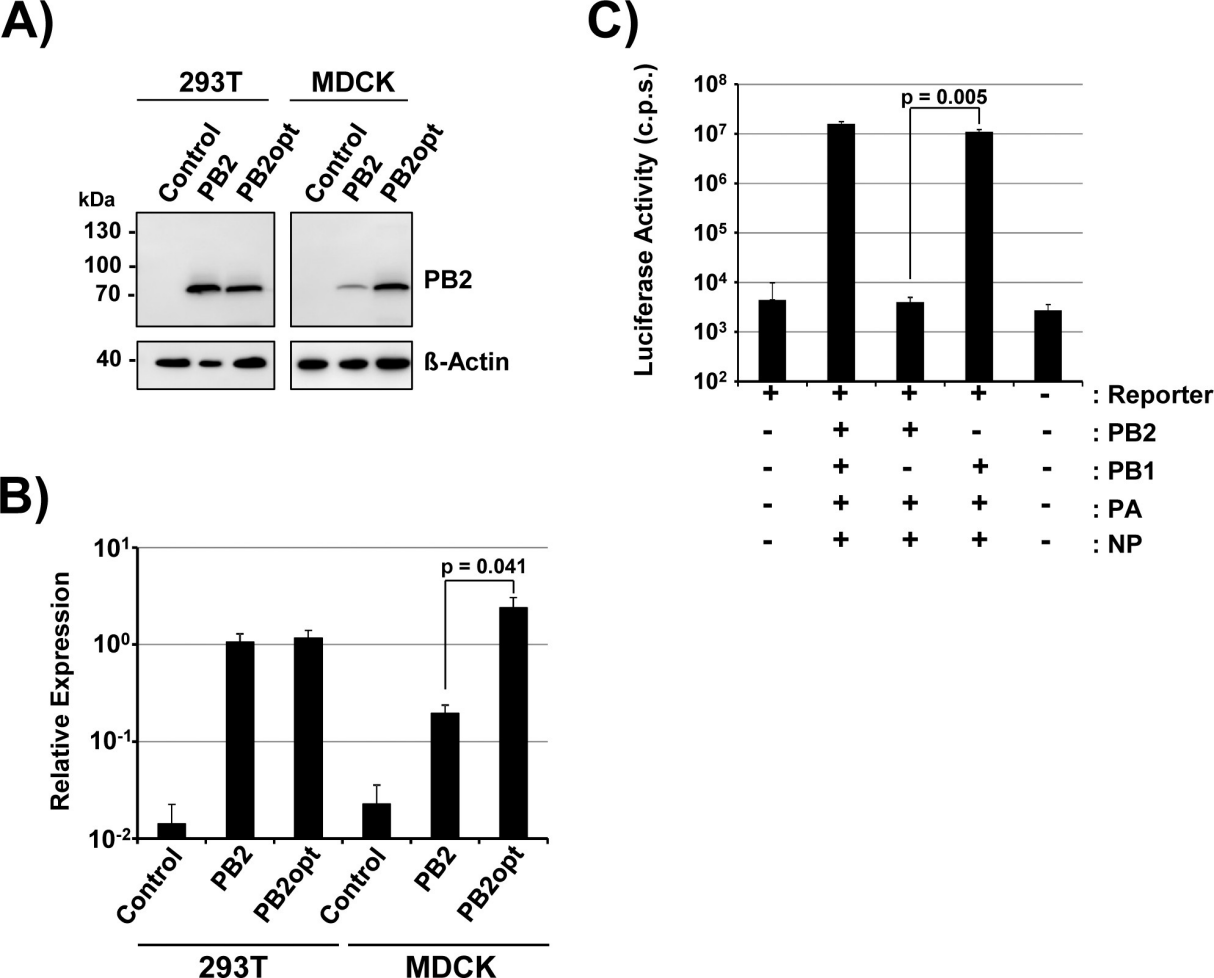
Stable expression of active PB2 protein in 293T and MDCK cells. (A) 293T and MDCK cells engineered to stably express PB2 or codon-optimized PB2 (PB2opt) were analyzed for PB2 expression by immunoblot using anti-PB2 antibody. Detection of beta-actin expression served as loading control. Similar results were obtained in four separate experiments. (B) The average of five experiments conducted as described for panel A and quantified via the ImageJ program is shown. Signals measured for PB2 or PB2opt were normalized against those measured for beta-actin. Error bars indicate standard error of the mean (SEM). Two tailed paired students t-test was used to assess statistical significance. (C) 293T cells stably expressing PB2 were cotransfected with plasmids encoding an IAV luciferase reporter segment and the indicated IAV proteins. Luciferase activities in cell lysates were determined at 24 h post transfection. The results of a representative experiment carried out with triplicate samples are shown. Error bars indicate standard deviation. Two tailed paired students t-test was used to assess statistical significance. Similar results were obtained in three separate experiments. C.p.s., counts per second.

We sought to determine whether DI-244 particles can be amplified in the absence of standard virus if producer cells are engineered to express PB2. For this, we first used retroviral transduction and selection antibiotics to generate 293T and MDCK cell lines stably expressing PB2. Immunoblot revealed that the cell lines obtained by selection expressed robust levels of PB2 (Fig 1A and Fig 1B). In order to analyze whether PB2 is functional in these cells, we employed a mini-replicon system, which measures the amplification of a firefly luciferase encoding IAV reporter segment upon coexpression of PB2, PB1, PA and NP [30]. We found that transfection of 293T-PB2 cells with a plasmid encoding the reporter segment alone yielded luciferase activity in the background range while cotransfection of PB2, PB1, PA and NP expression plasmids increased luciferase activity more than 1,000-fold (Fig 1C). Importantly, this increase was not observed when the PB1 plasmid was omitted while omission of the PB2 plasmid had no impact on reporter activity (Fig 1C). Thus, the PB2 protein stably expressed in 293T cells was functional. Unfortunately, similar studies in MDCK cells were not feasible due to the low transfectability of these cells.

### PB2 expression allows production of infectious DI-244 in the absence of standard virus

**Fig 2:**
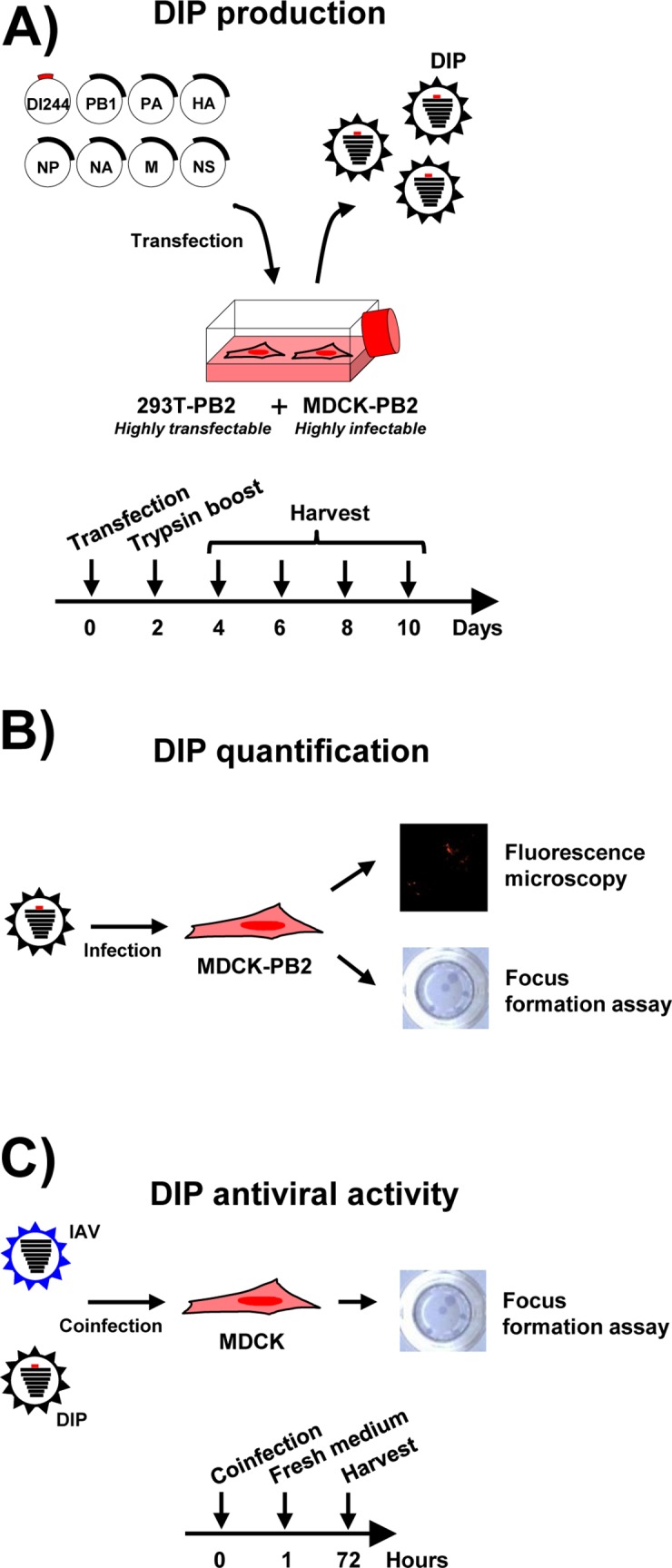
Schematic representation of the work-flow employed for DIP production and characterization. (A) For production of DIPs (DI-244-mScarlet-i), a coculture of 293T-PB2 and MDCK-PB2 cells was cotransfected with plasmids harboring DI-244-mScarlet-i and the wt IAV genomic segments two to eight. Subsequently, trypsin was added for HA activation and supernatants were harvested at the indicated time points. (B) For quantification of DIP production, MDCK-PB2 cells were inoculated with DIP containing supernatants and the number of red cells was counted or the number of foci was determined using focus formation assay. (C) For analysis of antiviral activity of DIPs, MDCK cells were coinfected with IAV wt and DIPs followed by focus formation assay.

**Fig 3:**
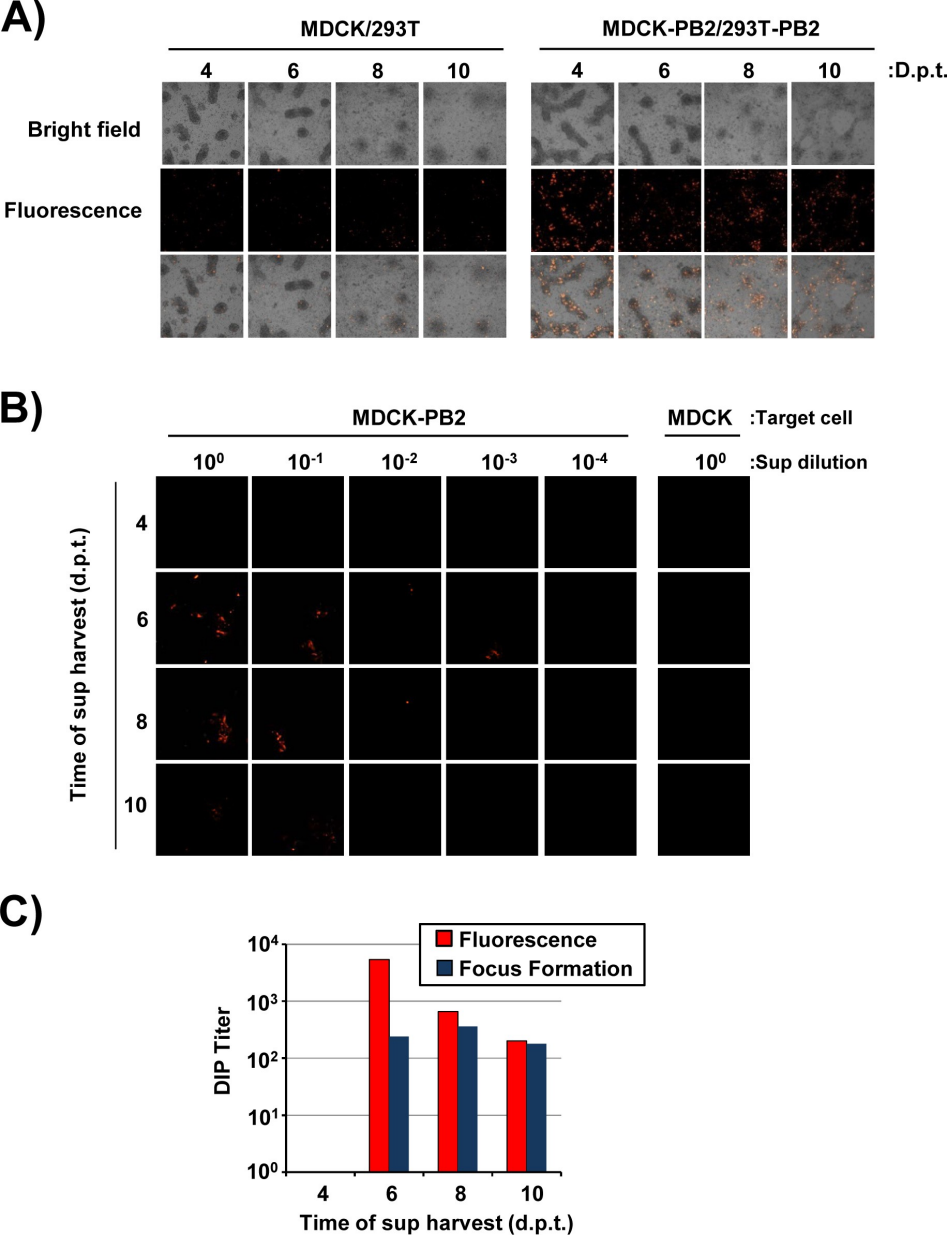
Production of DI-244 particles in PB2 expressing cell lines. (A) Cocultures of 293T-PB2/MDCK-PB2 cells were transfected with plasmids encoding wt IAV segments 2–8 and DI-244-mScarlet-i. The presence of red fluorescence at 4, 6, 8 and 10 days post transfection was analyzed using confocal microscopy. (B) MDCK control and MDCK-PB2 cells were infected with serially diluted DI-244 containing supernatants harvested at the indicated time points and produced as described in panel A followed by removal of supernatants and addition of methyl cellulose overlay. Thereafter, the presence of red fluorescent cells was analyzed at 72 h post infection using confocal microscopy. (C) The number of infected cells (as determined by red fluorescence) in panel B was quantified. In parallel, infection of cells was analyzed by focus formation assay and the number of foci quantified. The results of a representative experiment are shown in panels A-C and were confirmed in two separate experiments.

We next investigated whether the 293T-PB2 and MDCK-PB2 cells allowed the generation of DI-244 particles, using the experimental setup depicted in Fig 2A. In order to be able to visually inspect DI-244 production and spread, we generated a DI-244 variant that encodes for mScarlet-i, a red fluorescent protein [26]. Transfection of a mixture of 293T/MDCK cells with plasmids encoding IAV wt segments 2–8 jointly with a plasmid encoding DI-244-mScarlet-i resulted in occasional and moderate red fluorescence (Fig 3A). In contrast, frequent and prominent red fluorescence was observed in 293T-PB2/MDCK-PB2 cocultures (Fig 3A), indicating that the stably expressed PB2 promoted amplification of the DI-244-mScarlet-i DI RNA.

In order to examine whether amplification of the DI-244-mScarlet-i DI RNA resulted in the production of infectious DIPs, the supernatants of the transfected 293T-PB2/MDCK-PB2 cells were inoculated onto MDCK-PB2 cells (Fig 2B). As controls, the supernatants were also added to MDCK wt cells. Inoculation of MDCK-PB2 cells with supernatants from 293T-PB2/MDCK-PB2 cells resulted in infection of the target cells, as determined by expression of mScarlet-i (Fig 3B). The number of mScarlet-i-positive cells was concentration dependent and supernatants taken at 6 days post transfection from DIP producing cells contained the highest amount of infectivity (Fig 3B). Finally, no cells with prominent red fluorescence were detected under control conditions, indicating that DIPs were only infectious for MDCK-PB2 but not MDCK wt cells.

We next asked whether DI-244 production could be quantified by focus formation assay, which is based on detection of IAV antigens by antibody staining and is frequently employed to measure IAV infectivity. Moreover, we examined whether results obtained in the focus formation assay would match those obtained upon counting of foci based upon red fluorescence. Foci were observed in MDCK-PB2 but not in MDCK control cells, confirming that DIP infectivity requires PB2 expression in target cells. Quantification of DIP infectivity by focus formation assay revealed that maximum titers of roughly 1 x 10^3^ DIPs per ml were obtained and counting red fluorescent foci yielded roughly comparable results (Fig 3B and Fig 3C). Thus, expression of PB2 is sufficient for DI-244 production in the absence of helper virus but production efficiency is moderate.

### Codon optimization of PB2 allows increased PB2 expression and DIP production

**Fig 4:**
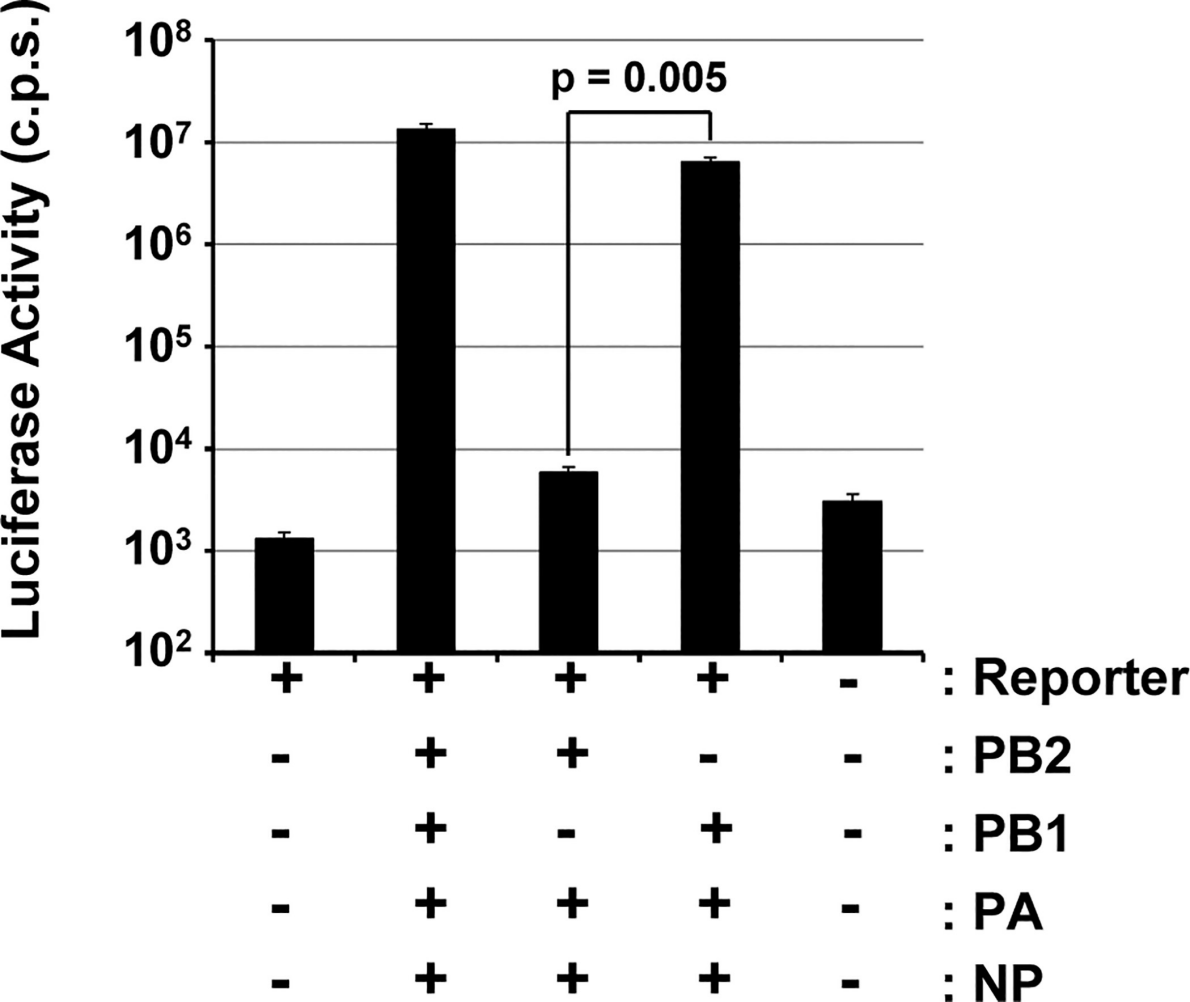
PB2opt stably expressed in 293T cells is active. 293T cells stably expressing PB2opt were cotransfected with plasmids encoding an IAV luciferase reporter segment and the indicated IAV proteins. Luciferase activities in cell lysates were determined at 24 h post transfection. The results of a representative experiment carried out with triplicate samples are shown. Error bars indicate standard deviation. Similar results were obtained in three separate experiments. Two tailed paired students t-test was used to assess statistical significance. C.p.s., counts per second.

DIP titers of 1 x 10^3^ particles per ml are low and may limit experimentation. Therefore, we next asked whether alteration of codon usage for PB2 expression might increase PB2 expression efficiency and DIP production. For this, we modified the codons in the PB2 expression plasmid (S1 Fig) to reflect codon preferences of human genes and IAV. As a second criterion for codon choice, we opted for maximal sequence difference between the A/PR/8/34-based sequence previously used for PB2 expression and the newly generated, optimized PB2 sequence (PB2opt), in order to prevent potential recombination events. 293T and MDCK cells were engineered to stably express PB2opt and immunoblot revealed that expression levels of PB2opt in MDCK but not 293T cells were higher than those obtained upon expression of non-codon-optimized PB2 (Fig 1A and Fig 1B). Moreover, growth of PB2opt cells was comparable to that of control cells and PB2opt expression was readily detectable after multiple passages, suggesting that expression was not associated with overt cytotoxicity. Finally, analysis of 293T-PB2opt cells in the mini-replicon assay showed that PB2opt supported IAV segment replication (Fig 4).

**Fig 5:**
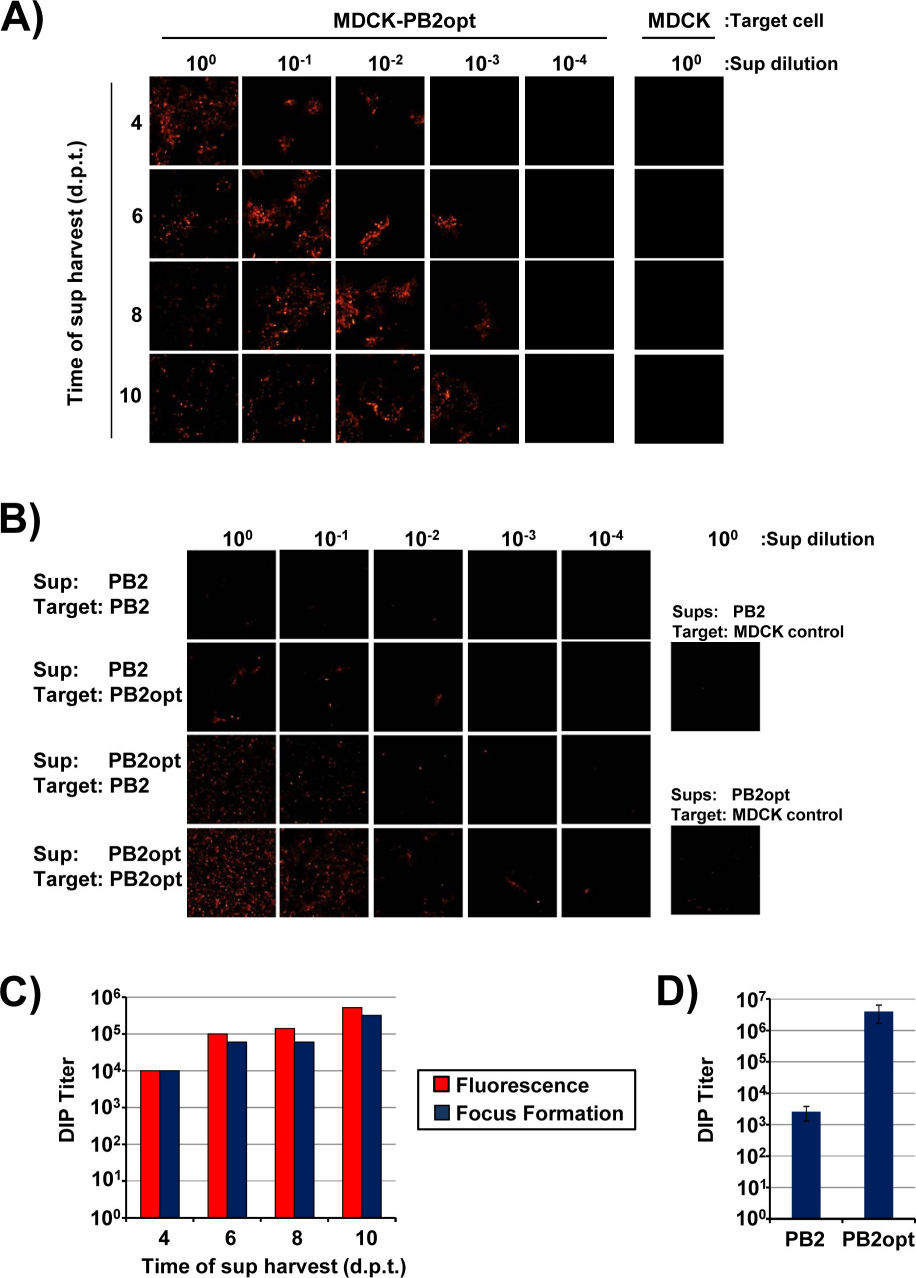
Codon optimization of PB2 results in increased DIP production. (A) DI-244 containing supernatants were produced in 293T-PB2opt/MDCK-PB2opt cells and harvested at the indicated time points as described for panel A of Fig 2. Subsequently, MDCK control and MDCK-PB2opt cells were infected with serially diluted DI-244 containing supernatants followed by removal of supernatants and addition of methyl cellulose overlay. Thereafter, the presence of red fluorescent cells was analyzed at 72 h post infection using confocal microscopy. (B) DI-244 supernatants produced in cells expressing PB2 or PB2opt were inoculated onto the indicated MDCK target cells as described for panel B and the presence of red fluorescent cells was analyzed at 72 h post infection using confocal microscopy. (C) The number of infected cells (as determined by red fluorescence) in panel A was quantified. In parallel, infection of cells was analyzed by focus formation assay and the number of foci was quantified. The results of a representative experiment are shown in panels A-C and were confirmed in two separate experiments. (D) The average of three (PB2) and six (PB2opt) independent experiments conducted as described for panel A is shown. Supernatants obtained at six days post transfection were analyzed, infection of cells was quantified by focus formation assay. Error bars indicate SEM.

Next, we examined whether PB2opt supports DIP production with higher efficiency than unmodified PB2. Efficient DI-244-mScarlet-i DI RNA amplification was observed in transfected PB2opt cells (not shown) and supernatants obtained from these cells were highly infectious for MDCK-PB2opt cells even when diluted 1:1,000 (Fig 5A). In contrast, the supernatants were not infectious for MDCK cells (Fig 5A). Moreover, a direct comparison of 293T-PB2/MDCK-PB2 and 293T-PB2opt/MDCK-PB2opt cells for production of infectious DIPs and for DIP amplification upon infection revealed that the PB2opt cells were more efficient. Thus, more red fluorescent cells were observed when supernatants from PB2 expressing cells were added to MDCK-PB2opt as compared to MDCK-PB2 cells (Fig 5B). Similarly, supernatants from PB2opt cells were more infectious for target MDCK-PB2opt cells as compared to MDCK-PB2 cells. In keeping with this observation, quantification of production of infectious DIPs by focus formation assay and counting of red fluorescent cells revealed that at least 80% of foci (identified by antibody staining) were positive for mScarlet-i, as expected, and that PB2opt cells produced up to 4 x 10^6^ infectious DIPs per ml and thereby exceeded titers obtained with PB2 cells (2,5 x 10^3^) by ~1,500-fold (Fig 5C and Fig 5D).

### DI-244 produced in the absence of standard virus exerts antiviral activity

**Fig 6:**
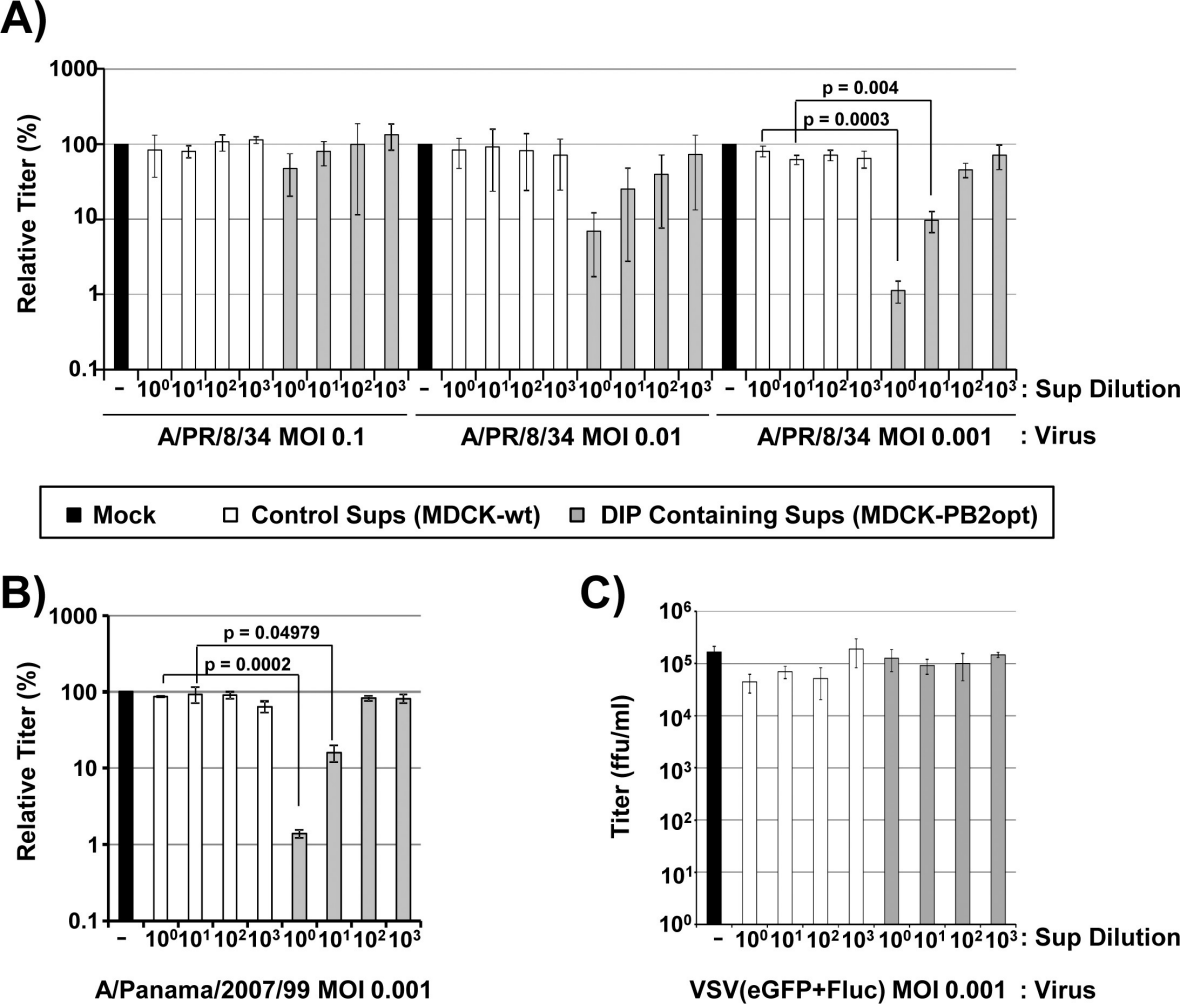
DI-244 produced in PB2opt expressing cell lines exerts anti-IAV activity. (A) Undiluted or 10-fold serially diluted DIP supernatants harvested from transfected 293T-PB2opt/MDCK-PB2opt cells or supernatants from transfected 293T/MDCK control cells were co-inoculated with A/PR/8/34 (H1N1) onto MDCK cells. Fresh medium was added at 1 h post infection and infectivity present in supernatants harvested at 72 h post infection was analyzed by focus formation assay. The average of three (MOI 0.1, MOI 0.01) and six (MOI 0.001), respectively, independent experiments is shown. Infection in the absence of supernatants was set as 100%. Error bars indicate standard error of the mean (SEM). Two tailed paired students t-test was used to assess statistical significance. (B) The experiment was carried out as described for panel A but A/Panama/2007/99 (H3N2) was used for infection. The average of three independent experiments is shown. Infection in the absence of supernatants was set as 100%. Error bars indicate SEM. Two tailed paired students t-test was used to assess statistical significance. (C) The experiment was carried out as for panel A but cells were infected with GFP-encoding VSV and supernatants were harvested for titration at 24 h post infection. The results of a single representative experiment conducted with triplicate samples are shown and were confirmed in two separate experiments.

DI-244 can inhibit spread of diverse IAVs and, likely via induction of interferon (IFN), may also inhibit spread of unrelated viruses [3, 4]. In order to investigate the antiviral activity of DI-244-mScarlet-i, we first analyzed whether DI-244-mScarlet-i produced in PB2opt cells interfered with the spread of a homologous IAV, A/PR/8/34, in MDCK cells (Fig 2C). For this, MDCK cells were coinfected with the indicated dilutions of DI-244 containing supernatants and A/PR/8/34 at an MOI of 0.1, 0.01 and 0.001 (Fig 6A). This resulted in IAV/DIP ratios of approximately 1:10 (undiluted DIP containing supernatants, IAV at MOI 0.1), 1:100 (undiluted DIP containing supernatants, IAV at MOI 0.01) and 1:1,000 (undiluted DIP containing supernatants, IAV at MOI 0.001), respectively. The supernatants from 293T/MDCK wt cells transfected with plasmids for DI-244 production were used as negative control. The control supernatants did not appreciably interfere with A/PR/8/34 infection while supernatants from PB2opt cells efficiently blocked IAV infection in a concentration dependent manner, with highest antiviral activity observed at an IAV/DIP ratio of 1:1,000 (Fig 6A). Specifically, infection efficiency relative to untreated virus (set as 100%) was 1 ± 0.5% in the presence of DIP containing supernatants at a dilution of 10^0^ and 93 ± 13% in the presence of control supernatants (average of six independent experiments). Moreover, DI-244 containing supernatants also inhibited infection by A/Panama/2007/99 (H3N2) in a concentration dependent manner (Fig 6B), in keeping with the concept that DI-244 exerts broad anti-IAV activity [3, 4]. Finally, DI-244 containing supernatants did not inhibit VSV infection (Fig 6C), indicating that DI-244 neither interfered with VSV genome replication nor altered viral control by a potential IFN response in MDCK cells. These results show that DI-244 produced in PB2opt expressing cells exerts potent anti-IAV activity.

## Discussion

The generation of DIPs in IAV infected cells has been recognized by von Magnus several decades ago [33] and DIPs hold promise as novel antiviral agents [3, 4]. However, exploitation of DIPs for antiviral therapy requires efficient production systems that do not depend on the presence of standard virus. Here, we report a DI-244 variant encoding a fluorescent protein that permits monitoring of DIP production. Moreover, we demonstrate that cells expressing PB2 allow generation of infectious DI-244 particles solely from plasmids and in the absence of standard virus. Finally, our study shows that DIPs produced in this system suppress spread of different IAV subtypes but not VSV in cell culture.

DI-244 particles and other DIPs have so far been amplified in cell culture or hen’s eggs in the presence of standard virus [3, 4, 17]. In addition, production of DI-244 particles from a plasmid system has been described [34, 35]. This approach relies on the transfection of plasmids for production of infectious IAV in conjunction with a plasmid containing the DI-244 segment and results in the co-production of DIPs and standard virus [34, 35]. Before DIP preparations produced in these systems can be used for experimentation, the remaining standard virus needs to be inactivated by UV light [18]. This approach builds on the preferential inactivation of standard virus relative to DIPs. Thus, a mutation in a gene essential for viral spread will abrogate infectivity of standard virus but may have no effect on DIP infectivity since the missing proteins will be complemented in trans in cells coinfected with DIPs and standard virus. However, controlling the efficiency of UV inactivation of standard virus is technically challenging. Moreover, the effect of UV light on DIP infectivity is difficult to determine and both issues may complicate large scale production of DIPs as well as interpretation of experimental data and animal trials. Thus, establishment of novel cell culture systems for DIP production in the absence of standard virus is an important task.

Our results show that cell lines expressing PB2 allow production and quantification of DI-244 particles solely from plasmids and in the absence of standard virus. This finding was not expected given that several reports indicate that PB2 expression alone is insufficient to allow robust spread of IAV variants with temperature sensitive mutations in the PB2 gene at non-permissive temperatures [20, 21]. Moreover, it has been suggested that PB2 expression might be associated with unwanted cytotoxic effects [4]. The present study suggests that up to 4 x 10^6^ DI-244 particles/ml can be produced in cells expressing codon optimized PB2, which roughly translates into production of 10 infectious DIPs per cell, and it can be speculated that efficiency of DIP production can be further increased by employing cell lines stably coexpressing PB1, PB2 and PA. Occasionally, weak fluorescence has been observed in DIP inoculated control cells. This is most likely attributable to low levels of DI-244 mRNA expression facilitated by PB2 protein associated with DI-244 vRNA present in the infecting DIPs. In contrast, no evidence for production of infectious IAV due to recombination between the DI-244 RNA and the RNA encoding for PB2 was obtained, as judged by bright field microscopy, immunofluorescence, focus formation assay and RT-PCR analysis, indicating that the DIP production system reported here is safe.

Quantification of DIP production so far relied on quantitative RT-PCR and hemagglutination assay [4, 8, 17], which do not provide information on particle infectivity. This limitation has been overcome by the present study which demonstrates that infectivity of DI-244 particles can be quantified using a standard technique, focus formation assay. The availability of this method should help comparing results obtained with different DI-244 preparations or other segment 1 DIPs and should thus advance the development of DIPs as antiviral agents. In this context, it is noteworthy that a IAV/DIP ratio of 1:1,000 resulted in the most prominent antiviral activity in our hands and a very similar ratio, 1:3,400 (as determined by estimations based on quantitative RT-PCR (DIP) and infectious units (IAV)), was previously reported to be minimally required to protect mice from severe influenza [4]. Thus, our study confirms and extends published work indicating that DIPs have to be provided in vast excess to exert antiviral activity. Whether sufficient numbers of DIPs can be delivered to the human respiratory tract and remain stable to provide protection against influenza for a prolonged time remains to be determined. In this context, one can speculate that an IAV:DIP ratio of less than 1:1,000 might be sufficient for antiviral activity in humans, since DIPs might exert direct antiviral activity by inhibiting IAV genome replication and induce the IFN system. Moreover, DIPs were reported to have a long residence time in the respiratory tract of mice and DIP-treated animals were found to still be protected at one week after treatment [4, 35]. Thus, DIP stability in the respiratory tract might not pose a major hurdle to the use of DIPs for influenza prevention and therapy in humans. Finally, it should be stated that reassortment of DIPs with IAV in coinfected cells is likely to occur. However, if DIPs based on the low pathogenic A/PR/8/34 or related viruses are used (like in the present study), such reassortment events should not result in viruses with increased transmissibility or virulence as compared to the wt virus.

It is believed that DI-244 can interfere with spread of diverse IAV in cell culture due to genome competition [3, 4]. Indeed, DI-244 produced in PB2opt cells exerted comparable antiviral activity against H1N1 and H3N2 IAV (no statistically significant differences), in keeping with H3N2 polymerase complexes being fully functional on H1N1 genomic segments [36]. This matches data published for DI-244 generated by use of standard virus [35] and demonstrates that DIPs produced in PB2 expressing cells are fully functional, although the activity of purified DIPs remains to be examined. DI-244 can also interfere with the spread of influenza B virus (IBV) and unrelated respiratory viruses in the infected host and this is thought to be due to induction of innate immune responses, particularly the IFN response [14, 16]. In contrast, DIP-mediated inhibition of IBV infection in cell culture is not observed, due to absence of genome competition [13, 14]. The absence of antiviral activity of DIPs against VSV confirms lack of genome competition. Moreover, it suggests that DIPs might not have modulated a potential IFN response in MDCK cells, although it should be noted that such a response might have been impeded due to the presence of trypsin in the culture medium [37].

Collectively, we report, to our knowledge, the first experimental system for production of DIPs without standard virus and for quantification of DIP infectivity, which should promote efforts to develop DIPs for antiviral therapy.

## Supporting information

S1 Fig Alignment of PB2 and codon optimized PB2. The nucleotide sequences of PB2-wt (PB2) and codon optimized PB2 (PB2opt) were aligned using the Clustal W algorithm of AlignX (Vector NTI). Divergent nucleotides are marked in black.
(PDF)
