# Supplementary material for: A system for production of defective interfering particles in the absence of infectious influenza A virus
Source: PLoS One. 2019 Mar 1;14(3):e0212757. doi: 10.1371/journal.pone.0212757 (PMC6396908; doi:10.1371/journal.pone.0212757)
Supplement: S1 Fig — The nucleotide sequences of PB2-wt (PB2) and codon optimized PB2 (PB2opt) were aligned using the Clustal W algorithm of AlignX (Vector NTI). Divergent nucleotides are marked in black. (PDF) [file pone.0212757.s001.pdf]

Legend to supplemental figure 1

S1 Fig. Alignment of PB2 and codon optimized PB2. The nucleotide sequences of PB2-wt (PB2) and codon optimized PB2 (PB2opt) were aligned using the Clustal W algorithm of AlignX (Vector NTI). Divergent nucleotides are marked in black.

```
         (1) 1       10        20        30        40            54
PB2      (1) ATGGAAAGAATAAAAGAACTACGAAATCTAATGTCGCAGTCTCGCACCCGCGAG
PB2opt   (1) ATGGAGCGGATCAAGGAGCTGCGGAACCTGATGAGCCAAAGCCGGACAAGAGAA

        (55) 55   60        70        80        90               108
PB2     (55) ATACTCACAAAAACCACCGTGGACCATATGGCCATAATCAAGAAGTACACATCA
PB2opt  (55) ATCCTGACCAAGACAACTGTTGATCACATGGCAATCATTAAAAAATATACCAGC

       (109) 109         120       130       140       150       162
PB2    (109) GGAAGACAGGAGAAGAACCCAGCACTTAGGATGAAATGGATGATGGCAATGAAA
PB2opt (109) GGCAGGCAAGAAAAAAATCCCGCTCTGCGAATGAAGTGGATGATGGCTATGAAG

       (163) 163    170       180       190       200            216
PB2    (163) TATCCAATTACAGCAGACAAGAGGATAACGGAAATGATTCCTGAGAGAAATGAG
PB2opt (163) TACCCCATCACCGCCGATAAACGAATCACCGAGATGATCCCCGAACGGAACGAA

       (217) 217          230       240       250       260        270
PB2    (217) CAAGGACAAACTTTATGGAGTAAAATGAATGATGCCGGATCAGACCGAGTGATG
PB2opt (217) CAGGGCCAGACACTGTGGTCCAAGATGAACGACGCAGGCAGCGATAGGGTTATG

       (271) 271     280       290       300       310           324
PB2    (271) GTATCACCTCTGGCTGTGACATGGTGGAATAGGAATGGACCAATAACAAATACA
PB2opt (271) GTGTCTCCACTCGCAGTTACCTGGTGGAACAGAAACGGCCCTATCACCAACACC

       (325) 325  330       340       350       360               378
PB2    (325) GTTCATTATCCAAAAATCTACAAAACTTATTTTGAAAGAGTCGAAAGGCTAAAG
PB2opt (325) GTGCACTACCCCAAGATATATAAGACCTACTTCGAGAGGGTGGAGCGACTGAAA

       (379) 379         390       400       410       420       432
PB2    (379) CATGGAACCTTTGGCCCTGTCCATTTTAGAAACCAAGTCAAAATACGTCGGAGA
PB2opt (379) CACGGCACATTCGGACCCGTGCACTTCCGGAATCAGGTGAAGATCAGGAGAAGG

       (433) 433    440       450       460       470            486
PB2    (433) GTTGACATAAATCCTGGTCATGCAGATCTCAGTGCCAAGGAGGCACAGGATGTA
PB2opt (433) GTGGATATCAACCCAGGCCACGCTGACCTGTCAGCAAAAGAAGCTCAAGACGTC
```

```
        (487) 487          500       510       520       530      540
PB2     (487) ATCATGGAAGTTGTTTTCCCTAACGAAGTGGGAGCCAGGATACTAACATCGGAA
PB2opt  (487) ATTATGGAGGTGGTGTTTCCCAATGAGGTTGGCGCAAGAATCCTGACCAGCGAG

        (541) 541      550       560       570       580           594
PB2     (541) TCGCAACTAACGATAACCAAAGAGAAGAAAGAAGAACTCCAGGATTGCAAAATT
PB2opt  (541) AGCCAGCTGACCATCACAAAGGAAAAAAAGGAGGAGCTGCAAGACTGTAAGATC

        (595) 595  600       610       620       630               648
PB2     (595) TCTCCTTTGATGGTTGCATACATGTTGGAGAGAGAACTGGTCCGCAAAACGAGA
PB2opt  (595) AGCCCACTCATGGTGGCCTATATGCTCGAACGGGAGCTTGTGAGAAAGACCAGG

        (649) 649        660       670       680       690          702
PB2     (649) TTCCTCCCAGTGGCTGGTGGAACAAGCAGTGTGTACATTGAAGTGTTGCATTTG
PB2opt  (649) TTTCTGCCTGTCGCAGGCGGCACTTCAAGCGTCTATATCGAGGTCCTCCACCTC

        (703) 703    710       720       730       740              756
PB2     (703) ACTCAAGGAACATGCTGGGAACAGATGTATACTCCAGGAGGGGAAGTGAGGAAT
PB2opt  (703) ACACAGGGCACCTGTTGGGAGCAAATGTACACCCCTGGCGGCGAGGTCCGAAAC

        (757) 757          770       780       790       800      810
PB2     (757) GATGATGTTGATCAAAGCTTGATTATTGCTGCTAGGAACATAGTGAGAAGAGCT
PB2opt  (757) GACGACGTGGACCAGTCACTCATCATCGCCGCCAGAAATATCGTCCGGAGGGCC

        (811) 811      820       830       840       850           864
PB2     (811) GCAGTATCAGCAGATCCACTAGCATCTTTATTGGAGATGTGCCACAGCACACAG
PB2opt  (811) GCCGTGTCTGCTGACCCTCTTGCCAGCCTGCTCGAAATGTGTCATTCAACTCAA

        (865) 865  870       880       890       900               918
PB2     (865) ATTGGTGGAATTAGGATGGTAGACATCCTTAGGCAGAACCCAACAGAAGAGCAA
PB2opt  (865) ATCGGCGGCATCAGAATGGTGGATATTTTGAGACAAAATCCCACCGAGGAACAG

        (919) 919        930       940       950       960          972
PB2     (919) GCCGTGGATATATGCAAGGCTGCAATGGGACTGAGAATTAGCTCATCCTTCAGT
PB2opt  (919) GCAGTTGACATCTGTAAAGCCGCCATGGGCCTCAGGATCTCAAGCAGCTTTAGC
```

```
         (973)  973    980       990       1000      1010            1026
PB2      (973)  TTTGGTGGATTCACATTTAAGAGAACAAGCGGATCATCAGTCAAGAGAGAGGAA
PB2opt   (973)  TTCGGCGGCTTTACCTTCAAAAGGACCTCAGGCTCTAGCGTGAAACGCGAAGAG

        (1027)  1027         1040      1050      1060      1070      1080
PB2     (1027)  GAGGTGCTTACGGGCAATCTTCAAACATTGAAGATAAGAGTGCATGAGGGATAT
PB2opt  (1027)  GAAGTCCTGACCGGAAACTTGCAGACCCTCAAAATCAGGGTCCACGAAGGCTAC

        (1081)  1081     1090      1100      1110      1120          1134
PB2     (1081)  GAAGAGTTCACAATGGTTGGGAGAAGAGCAACAGCCATACTCAGAAAAGCAACC
PB2opt  (1081)  GAGGAATTTACCATGGTCGGAAGGAGGGCCACCGCAATCCTGAGGAAGGCCACA

        (1135)  1135 1140      1150      1160      1170                1188
PB2     (1135)  AGGAGATTGATTCAGCTGATAGTGAGTGGGAGAGACGAACAGTCGATTGCCGAA
PB2opt  (1135)  AGAAGGCTCATCCAACTCATTGTCTCAGGCAGGGATGAGCAAAGCATCGCAGAG

        (1189)  1189       1200      1210      1220      1230          1242
PB2     (1189)  GCAATAATTGTGGCCATGGTATTTTCACAAGAGGATTGTATGATAAAAGCAGTC
PB2opt  (1189)  GCCATCATCGTCGCAATGGTGTTCAGCCAGGAAGACTGCATGATCAAGGCCGTG

        (1243)  1243   1250      1260      1270      1280                1296
PB2     (1243)  AGAGGTGATCTGAATTTCGTCAATAGGGCGAATCAACGATTGAATCCTATGCAT
PB2opt  (1243)  CGGGGCGACCTCAACTTTGTGAACAGAGCCAACCAGAGACTCAACCCAATGCAC

        (1297)  1297         1310      1320      1330      1340      1350
PB2     (1297)  CAACTTTTAAGACATTTTCAGAAGGATGCGAAAGTGCTTTTTCAAAATTGGGGA
PB2opt  (1297)  CAGCTGCTGCGGCACTTCCAAAAAGACGCCAAGGTCCTGTTCCAGAACTGGGGC

        (1351)  1351     1360      1370      1380      1390          1404
PB2     (1351)  GTTGAACCTATCGACAATGTGATGGGAATGATTGGGATATTGCCCGACATGACT
PB2opt  (1351)  GTCGAGCCCATTGATAACGTCATGGGCATGATCGGCATCCTCCCTGATATGACC

        (1405)  1405 1410      1420      1430      1440                1458
PB2     (1405)  CCAAGCATCGAGATGTCAATGAGAGGAGTGAGAATCAGCAAAATGGGTGTAGAT
PB2opt  (1405)  CCTTCTATTGAAATGAGCATGAGGGGCGTCCGGATTTCTAAGATGGGCGTTGAC
```

```
(1459)     1459       1470      1480      1490      1500        1512
PB2 (1459)    GAGTACTCCAGCACGGAGAGGGTAGTGGTGAGCATTGACCGTTTTTTGAGAATC
PB2opt (1459) GAATATAGCTCAACCGAAAGAGTGGTTGTCTCAATAGATAGGTTCCTCAGGATT

(1513)     1513   1520      1530      1540      1550            1566
PB2 (1513)    CGGGACCAACGAGGAAATGTACTACTGTCTCCCGAGGAGGTCAGTGAAACACAG
PB2opt (1513) AGAGATCAGAGGGGCAACGTGCTGTTGAGCCCTGAAGAAGTGTCTGAGACTCAA

(1567)     1567         1580      1590      1600      1610        1620
PB2 (1567)    GGAACAGAGAAACTGACAATAACTTACTCATCGTCAATGATGTGGGAGATTAAT
PB2opt (1567) GGCACCGAAAAGCTCACCATCACATATAGCAGCTCCATGATGTGGGAAATCAAC

(1621)     1621     1630      1640      1650      1660            1674
PB2 (1621)    GGTCCTGAATCAGTGTTGGTCAATACCTATCAATGGATCATCAGAAACTGGGAA
PB2opt (1621) GGCCCAGAGAGCGTCCTCGTGAACACATACCAGTGGATTATCCGGAATTGGGAG

(1675)     1675 1680      1690      1700      1710                1728
PB2 (1675)    ACTGTTAAAATTCAGTGGTCCCAGAACCCTACAATGCTATACAATAAAATGGAA
PB2opt (1675) ACAGTGAAGATCCAATGGAGCCAAAATCCAACCATGCTGTATAACAAGATGGAG

(1729)     1729       1740      1750      1760      1770        1782
PB2 (1729)    TTTGAACCATTTCAGTCTTTAGTACCTAAGGCCATTAGAGGCCAATACAGTGGG
PB2opt (1729) TTCGAGCCCTTCCAAAGTCTGGTGCCCAAAGCAATCAGGGGACAGTATTCTGGC

(1783)     1783   1790      1800      1810      1820            1836
PB2 (1783)    TTTGTAAGAACTCTGTTCCAACAAATGAGGGATGTGCTTGGGACATTTGATACC
PB2opt (1783) TTCGTGCGGACCCTCTTTCAGCAGATGAGAGACGTCCTGGGCACCTTCGACACA

(1837)     1837         1850      1860      1870      1880        1890
PB2 (1837)    GCACAGATAATAAAACTTCTTCCCTTCGCAGCCGCTCCACCAAAGCAAAGTAGA
PB2opt (1837) GCCCAAATCATCAAGCTGCTGCCATTTGCCGCTGCCCCTCCTAAACAGAGCAGG

(1891)     1891     1900      1910      1920      1930            1944
PB2 (1891)    ATGCAGTTCTCCTCATTTACTGTGAATGTGAGGGGATCAGGAATGAGAATACTT
PB2opt (1891) ATGCAATTTAGCAGCTTCACCGTCAACGTCAGAGGCAGCGGGATGCGGATCCTC
```

	(1945)	1945 1950 1960 1970 1980 1998
PB2	(1945)	GTAAGGGGCAATTCTCCTGTATTCAACTATAACAAGGCCACGAAGAGACTCACA
PB2opt	(1945)	GTGAGAGGAAACAGCCCCGTGTTTAATTACAATAAAGCAACCAAAAGGCTGACC
	(1999)	1999 2010 2020 2030 2040 2052
PB2	(1999)	GTTCTCGGAAAGGATGCTGGCACTTTAACTGAAGACCCAGATGAAGGCACAGCT
PB2opt	(1999)	GTGCTGGGGAAAGACGCCGGAACACTGACCGAGGATCCCGACGAGGGAACCGCA
	(2053)	2053 2060 2070 2080 2090 2106
PB2	(2053)	GGAGTGGAGTCCGCTGTTCTGAGGGATTCCTCATTCTGGGCAAAGAAGACAAG
PB2opt	(2053)	GGCGTCGAATCTGCCGTGCTCAGAGGCTTTCTGATCCTCGGAAAGGAGGATAAA
	(2107)	2107 2120 2130 2140 2150 2160
PB2	(2107)	AGATATGGGCCAGCACTAAGCATCAATGAACTGAGCAACCTTGCGAAAGGAGAG
PB2opt	(2107)	AGGTACGGCCCTGCTCTGTCCATAAACGAGTTGTCAAATCTGGCCAAGGGCGAA
	(2161)	2161 2170 2180 2190 2200 2214
PB2	(2161)	AAGGCTAATGTGCTAATTGGGCAAGGAGACGTGGTGTTGGTAATGAAACGGAAA
PB2opt	(2161)	AAAGCCAACGTCCTGATCGGACAGGGCGATGTCGTCCTCGTCATGAAGAGAAAG
	(2215)	2215 2220 2230 2240 2250 2268
PB2	(2215)	CGGGACTCTAGCATACTTACTGACAGCCAGACAGCGACCAAAAGAATTCGGATG
PB2opt	(2215)	AGAGATAGCTCAATCCTGACCGATTCACAAACCGCCACAAAGAGGATCAGAATG
	(2269)	2269 2277
PB2	(2269)	GCCATCAAT
PB2opt	(2269)	GCAATTAAC
